# Association of Serum C-Reactive Protein Level and Treatment Duration in Acute Cholecystitis Patients Treated Conservatively

**DOI:** 10.7759/cureus.22146

**Published:** 2022-02-12

**Authors:** Selahattin Vural, Ismail Aydin, Tugrul Kesicioglu

**Affiliations:** 1 General Surgery, Giresun University Faculty of Medicine, Giresun, TUR

**Keywords:** prognosis, medical, treatment, crp, acute cholecystitis

## Abstract

Background: Acute cholecystitis (AC) is one of the most common gastrointestinal diseases that require hospitalization and surgical treatment. The treatment of the disease depends upon the severity of the disease and the patients’ medical status.

Objective: In this study, we aimed to investigate if there is an association between the serum C-reactive protein (CRP) value and treatment response and the duration and length of hospital stay in AC patients who are treated conservatively.

Methodology: The medical records of all patients with the diagnosis of AC who were treated with conservative management were included in the study. The demographic and laboratory data including CRP level at first admission to hospital, length of hospital stay, and complications during the conservative treatment were obtained from the patients’ records. Patients were divided into two groups according to the treatment response and length of hospital stay. Group 1 patients were defined as patients who responded to the medical treatment in less than three days, and Group 2 patients were defined as patients who did not respond to the medical treatment in three days and stayed at the hospital for more than three days.

Results: We identified 101 patients with AC treated medically. Mean age (51.3 ± 16.3, 59.5 ± 15.7; p = 0.013), total leukocyte count (11.8 ± 4.4, 8.2 ± 2.8; p = 0.0005), and CRP value (19.3 ± 13.9, 9.6 ± 5.2; p = 0.0003) were higher in Group 2 compared to Group 1. Correlation analyses demonstrated a significant positive association between the length of hospital stay, total leukocyte count (r = 0.35; p = 0.0002), and CRP value (r = 0.59; p = 0.0004).

Conclusion: We found that CRP level is associated with treatment duration and hospital stay in AC patients. However, large-scale, prospective further studies are needed to confirm our results and to determine whether CRP levels can be used to discriminate which patient would benefit from medical treatment.

## Introduction

Acute cholecystitis (AC) defined by right upper quadrant pain, presence of Murphy’s sign, fever, elevated white blood cell count (WBC), and C-reactive protein (CRP) is one of the most common gastrointestinal diseases that require hospitalization and surgical treatment [1]. Obstruction of the bile duct mostly due to a gallstone or a tumor leads to biliary stenosis and results in inflammation and infection in the biliary system. Delay in treatment causes progression to severe disease, which is generally defined as inflammation, empyema, gangrene or perforation of gallbladder, adhesions, or difficulty in dissecting Calot’s triangle [2]. The treatment of AC depends on the severity of the disease and the patient’s general status [3]. Cholecystectomy which is mostly performed by laparoscopy or conservative treatment is considered the treatment of choice in patients presenting with AC [4,5].

CRP is a nonspecific acute-phase reactant protein synthesized in the liver, which is used as a systemic marker for inflammation. It correlates with the severity of infection and inflammation in most of the acute inflammatory diseases [6]. In recent studies, CRP is also found to be associated with the severity of AC [7].

Although there are studies showing an association between serum inflammation markers and severity of AC, there is no data about the serum CRP level and the treatment duration and length of the hospital stay of AC patients treated with conservative management. The aim of this study was to determine if clinical variables and serum inflammatory markers such as WBC and CRP values in the first admission of patients with AC are related to the treatment response and the duration and length of the hospital stay.

## Materials and methods

This case-control study was conducted at Giresun University, Prof Dr. A. İlhan Özdemir Education and Research Hospital in Turkey. The medical records of all patients presenting with abdominal pain to the emergency clinic between 2015 and 2018 were evaluated, and patients with a diagnosis of AC and those who were treated with conservative management were included in the study. The institutional review board of Ordu Üniversitesi Klinik Araştırmalar Etik Kurul approved our study (Approval number: 2020/198).

The diagnosis of AC was defined as the presence of abdominal pain in the right upper quadrant and positive Murphy’s sign with systemic signs of inflammation (fever and/or shaking chills or laboratory data as evidence of inflammatory response) or cholestasis (jaundice or laboratory data as abnormal liver function tests) and possibly with radiologic findings as biliary dilatation or evidence of the etiology on imaging (stricture, stone, stent, etc.) according to the Tokyo Guideline (TG) 2018 [8]. The severity of the disease was assessed with the severity grading criteria of TG 2018. Patients who managed with early and urgent cholecystectomy, Grade 3 disease, and malignancy, were excluded from the study.

Patients’ demographic data including age, body mass index (BMI), sex, comorbidities (diabetes mellitus, hypertension, thyroid disease, and romatological disease), and the laboratory data of routine serum hemoglobin level, WBC, alanine aminotransferase (ALT), aspartate aminotransferase (AST), and CRP level at first admission to the hospital were obtained from the patient records. We also recorded the length of hospital stay during treatment and the complications during conservative treatment that were defined as any event requiring additional treatment.

The standard conservative treatment regimen was intravenous fluid and electrolyte infusion, electrolyte correction, and antimicrobial therapy with a combination of intravenous metronidazole and cephalosporin with analgesics in the study population during the study period, and if the patients’ physical status was appropriate for surgery, cholecystectomy was offered three months after the first AC attack.

The Statistical Package for the Social Sciences (SPSS) program version 15.0 (SPSS Inc., Chicago, IL) was used for analysis. The results were presented as means ± standard deviation (SD) values. Kolmogorov-Smirnov test was used for normality of data distribution and variance homogeneity. The parameters with a normal distribution were compared between the groups by the student’s t-test. Parameters with non-normal distribution were compared between the groups by non-parametric tests such as the Mann-Whitney U test or the Fisher’s exact test.

Correlation analyses between parameters were made by Pearson’s or Spearman’s correlation tests depending on the distribution of data; a p-value of <0.05 was considered statistically significant.

## Results

A total of 108 patients diagnosed with cholecystitis at first hospital admission and treated with conservative treatment during the study period were included in the study. Conservative treatment included bowel rest and intravenous administration of broad-spectrum antibiotics and fluids. Seven patients showed worsening of clinical signs and laboratory results during follow-up, and emergency cholecystectomy was performed and excluded from the study. A total of 101 patients (93.5%) showed resolution of symptoms and were discharged successfully.

In our study population, the mean age was 56.0 ± 16.4, 56 patients (55.4%) were men, and 45 (44.6%) were women. The mean length of hospital stay was 5.5 ± 3.7 days, and the mean CRP value was 15.2 ± 12.06. There was no mortality in our study population. Demographic data and laboratory test results of our study population were shown in Table 1.

**Table 1:** Demographic and laboratory characteristics of the study population Data is expressed as mean ± SD. BMI: Body mass index; WBC: white blood cells; ALT: alanine aminotransferase; AST: aspartate aminotransferase; CRP: C-reactive protein.

Variables	n
Age (y), mean (SD)	56.0 ± 16.4
Sex, n (%)	
Male	56 (55.4)
Female	45 (44.6)
Comorbidity	
Yes	19 (18.8)
No	82 (81.2)
BMI (kg/m^2^)	27.6 ± 3.3
Hemoglobin (gr/dl)	13.4 ± 1.8
WBC x 10^3^ mL	10.3 ± 4.2
Total bilirubin (mg/dl)	1.46 ± 1.30
ALT (IU/L)	51.7 ± 50.2
AST (IU/L)	57.9 ± 62.5
Amylase	58.5 ± 28.9
CRP (mg/dL)	15.2 ± 12.0
Length of hospital stay (d)	5.5 ± 3.7

Patients were divided into two groups according to treatment response and the length of hospital stay. We defined Group 1 patients as patients who responded to medical treatment in less than three days and stayed at the hospital for one to three days, and Group 2 patients were defined as patients who did not respond to the medical treatment in three days and stayed at the hospital for more than three days. Table 2 summarizes the comparative analysis of Group 1 and Group 2 patients in terms of demographic, clinical, and laboratory data at the first hospital admission. Mean age (51.3 ± 16.3, 59.5 ± 15.7; p = 0.013), total leukocyte count (11.8 ± 4.4, 8.2 ± 2.8; p = 0.0005), and CRP value (19.3 ± 13.9, 9.6 ± 5.2; p = 0.0003) were higher in Group 2 patients compared to Group 1 patients.

**Table 2:** Demographic and laboratory characteristics of Group 1 and Group 2 patients Data is expressed as mean ± SD. BMI: Body mass index; WBC: white blood cells; ALT: alanine aminotransferase; AST: aspartate aminotransferase; CRP: C-reactive protein.

Variables	Group 1 (n = 43)	Group 2 (n = 58)	P-value
Age (y), mean (SD)	51.3 ± 16.3	59.5 ± 15.7	0.013
Sex, n (%)			
Male	27 (62.8)	30 (51.7)	0.22
Female	16 (37.2)	28 (48.3)	
Comorbidity, n (%)			
Yes	7 (16.3)	12 (20.7)	0.38
No	36 (83.7)	46 (79.3)	
BMI (kg/m^2^)	27.1 ± 2.7	27.5 ± 3.6	0.23
Hemoglobin (gr/dl)	13.1 ± 1.9	13.5 ± 1.7	0.28
WBC x 10^3^ mL	8.2 ± 2.8	11.8 ± 4.4	0.0005
Total bilirubin(mg/dl)	1.17 ± 0.8	1.67 ± 1.5	0.06
ALT (U/L)	37.1 ± 28.3	48.6 ± 32.9	0.07
AST (U/L)	39.8 ± 34.0	51.0 ± 43.9	0.17
Amylase	58.1 ± 23.7	58.8 ± 32.5	0.89
CRP (mg/dL)	9.6 ± 5.2	19.3 ± 13.9	0.0003
Length of hospital stay (d)	2.5 ± 1.07	7.6 ± 3.0	0.0005

**Figure 1:**
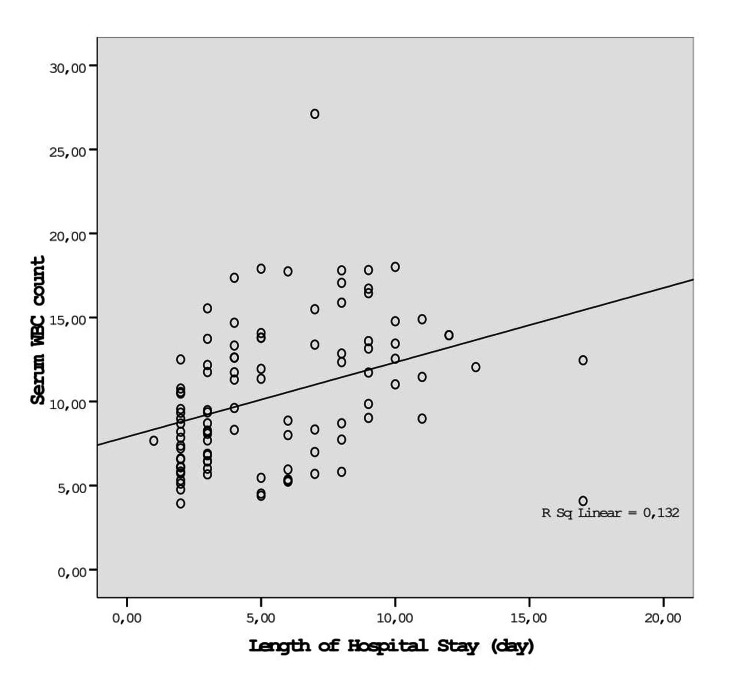
Correlation between the serum WBC level and length of hospital stay in patients with AC (p = 0.0004) WBC: White blood cells; AC: acute cholecystitis.

**Figure 2:**
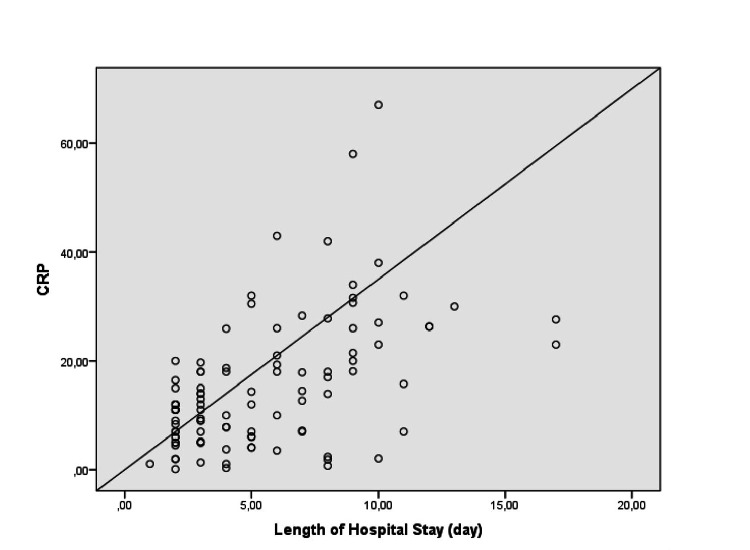
Correlation between the serum CRP level and length of hospital stay in patients with AC (p = 0.0002) CRP: C-reactive protein; AC: acute cholecystitis.

To evaluate a possible association between the serum total leukocyte count, CRP value, and length of hospital stay, we used bivariate correlation analyses. Correlation analyses demonstrated a significant positive association between the length of hospital stay and total leukocyte count (r = 0.35; p = 0.0002) and CRP value (r = 0.59; p = 0.0004) (Figures 1, 2).

## Discussion

In this study, we found that serum inflammation markers such as WBC and CRP at the first hospital admission were significantly lower in patients who respond to medical treatment earlier, and we have reported a positive and significant correlation between the serum CRP and WBC levels and the length of hospital stay in patients presenting with mild and moderate AC managed with conservative treatment. To the best of our knowledge, this is a rare study that evaluated and demonstrated an association between the CRP level and the length of hospital stay in AC with conservative treatment.

AC is a common disease with a high socio-economic impact. It is the sixth most common gastrointestinal disease diagnosed in emergency clinics and the second most common cause of hospitalization in the United States [9]. When AC is diagnosed, clinical management depends on the disease severity and patient’s status [5]. Laparoscopic cholecystectomy, percutaneous cholecystostomy, or conservative treatment are treatment modalities for patients with AC [10]. While cholecystectomy in patients with AC prevents the later recurrence of the disease, it has an approximately 15% complication rate. Conservative treatment prevents surgical complications; however, it may cause recurrent disease, or living with the gallbladder may cause progression to severe disease [11]. Although early laparoscopic cholecystectomy is recommended if the patient is suitable for surgery in Grade 1 disease in TG 2018 [5], there is a wide variation due to patient- or hospital-related factors in the management of patients presenting with AC worldwide.

Agrawal et al. showed 100% treatment responses with antibiotic treatment in patients with mild AC in 25 patients [12]. In another study, Gutt et al. found 92% treatment responses in their study [13]. Recently, Loozen et al. reported that 87% of patients with AC responded to the conservative treatment that included bowel rest and broad-spectrum antibiotics without the need for surgery or other intervention in a systematic review [11]. In our study, we found that 93.5% of our patients responded to medical treatment and were discharged successfully similar to the literature.

TG defined the severity of AC as Grades 1 to 3 according to clinical findings, physical examination, laboratory tests, and imaging methods. In Grade 1 disease, there are mild inflammatory changes in the gallbladder; in Grade 2 disease, there is a moderate inflammation without organ dysfunction; however, in Grade 3 disease, there is a severe inflammation with organ dysfunction [8]. The determination of the severity of disease in patients with AC in the first admission is important for choosing the treatment modality and determining the prognosis [14]. CRP is used only as a diagnostic criterion but not for severity assessment in this guideline.

CRP is an acute-phase reactant synthesized in the liver that increases in inflammatory diseases [6], and when levels to 10 mg/L are usually thought to be clinically insignificant, levels of 100 mg/L and higher are considered to be associated with tissue necrosis [15,16]. Studies in the recent literature have shown that increased systemic inflammatory markers are associated with severity and poor prognosis with many types of diseases such as cancer [17], acute appendicitis [18], acute heart failure [19], and sepsis [20].

Several studies also showed that serum CRP level was associated with AC [21,22] and found to be a predictive factor in the assessment of the severity of the disease [7,23]. Mok et al. observed that patients with gangrenous cholecystitis had a significantly higher CRP value, and CRP level > 200 mg/dL was found to be having a 50% positive and 100% negative predictive value for gangrenous cholecystitis with 100% sensitivity and 87.9% specificity [22]. Nikfarjam et al. reported that CRP value > 94 mg/L is a predictive factor for gangrenous cholecystitis [7]. Sato et al. found that serum neutrophil-lymphocyte ratio (NLR) and CRP/albumin ratio are significantly elevated in patients with AC with Grade 2 and Grade 3 diseases, and these markers could independently predict the Grade 2 and Grade 3 diseases [24]. Gurbulak et al. showed that the serum CRP level was a strong predictor in classifying different grades of the disease according to TG 13 and reported the cutoff values of CRP to be 7.065 mg/dl with 75.5% sensitivity and 96.5% specificity in patients with Grade 2 disease and 19.895 mg/dl with 73.9% sensitivity and 75.5% specificity in patients with Grade III disease, respectively [25]. Beliaev et al. evaluated the serum CRP level and NLR as a marker for the diagnosis of AC and prediction of the disease severity based on pathological findings, and they confirmed that CRP and NLR were superior to WBC in discriminative ability [1,26].

Although there are studies showing an association between the serum inflammatory markers such as CRP and WBC and disease severity in AC patients in the literature, there is no data about these markers and their relationship with the medical treatment response, treatment duration, and length of the hospital stay. In this study, we found that WBC and CRP levels are significantly associated with the treatment duration in AC patients with conservative treatment. The limitation of this study was its retrospective design and a low number of patients.

## Conclusions

In conclusion, although TG 18 recommends early laparoscopic cholecystectomy in patients with AC if the patient is suitable for surgery, conservative treatment can be seen as an option in the treatment modality of these patients. We found that CRP level is associated with the treatment duration and hospital stay in AC patients. However, our study is a retrospective and single-center study with relatively small sample size; therefore, large-scale, prospective, further studies are needed to confirm our results and to determine if CRP levels can be used to discriminate which patient would benefit from medical treatment.
